# Supplementary material for: Definition of a New HLA B*52-Restricted Rev CTL Epitope Targeted by an HIV-1-Infected Controller
Source: Viruses. 2023 Feb 18;15(2):567. doi: 10.3390/v15020567 (PMC9959870; doi:10.3390/v15020567)
Supplement: Supplementary file 1 [file viruses-15-00567-s001.zip › viruses-2173752-supplementary.pdf]

viruses

Supplementary Materials

Supplementary Table S1. Characteristics of study participants

Supplementary Table S2. Rev-RI8 recognition, immunological and virological parameters in study participants.

Supplementary Table S3. Sequence variants of the RI8 epitope within different HIV-1 subtypes.

Supplementary Table S4. Analysis of sequence variation within the RI8 epitope.

Supplementary Table S1. Characteristics of study participants

Patients	Age	Gender	Current CD4	Current CD8	Current viral load	Months on ART[2]	CDC-Stage
#1[1]	60	m	928	439	<20	0	A1
#2	41	m	838	645	<20	64	B1
#3	40	m	351	594	<20	27	B2
#4	37	m	714	438	<20	84	A2
#5	54	m	929	422	<20	119	A2
#6	52	m	1434	717	<20	135	A1
#7	46	f	723	942	<20	128	C3
#8	37	f	1097	627	<20	27	A1
#9	50	f	952	328	<20	65	B2
#10	48	f	1295	569	<20	315	B2
#11	44	m	548	344	<20	60	A1
#12	53	m	905	1149	<20	164	A3
#13	55	f	386	772	<20	42	C3
#14	47	f	395	997	40	3	B2
#15	52	m	1096	1388	<20	127	C3

[1] Controller #1 at the day of the ELISPot analysis with freshly isolated PBMCs (Figure 1),
[2] ART = antiretroviral therapy, m=male, f=female.

Supplementary Table S2. Rev-RI8 recognition, immunological and virological parameters in study participants.

Patients	RI8[1]	Current CD4	Current CD8	Current viral load	Pre-ART CD4	Pre-ART CD8	Pre-ART viral load
#1[2]	y	928	439	<20	928[3]	439[3]	<20[3]
#2	y	838	645	<20	720	1009	7600
#3	y	351	594	<20	329	1898	230000
#4	y	714	438	<20	483	1435	57500
#5	y	929	422	<20	263	n/a[2]	26000
#6	y	1434	717	<20	809	809	35000
#7	n	723	942	<20	5	940	140000
#8	n	1097	627	<20	39	542	2500
#9	n	952	*328*	<20	319	663	7000
#10	n	1295	569	<20	336	700	n/a[4]
#11	n	548	344	<20	517	818	291650
#12	n	905	1149	<20	108	n/a[4]	270000
#13	n	386	772	<20	16	380	560000
#14	n	395	997	40	254	863	140000
#15	n	1096	1388	<20	192	n/a[4]	560000

[1] RI8: Recognition of Rev-RI8 peptide: y=yes, n=no, [2] controller #1 at the day of the ELISPOT analysis with freshly isolated PBMCs (Figure 1), [3] same values as current values as #1 was untreated. [4] data not available. Pre-ART: last value before start of antiretroviral treatment. Statistical analysis by Mann-Whitney-U-Test revealed significant higher pre-ART CD4 counts in subjects with recognition of Rev-RI8 in comparison to subjects without recognition of Rev-RI8 (p=0.012 with inclusion subject #1 and p= 0,0290, if #1 is omitted). There were no significant differences between Rev-RI8 responders and non-responders regarding current CD4 counts, current and pre-ART CD8 counts and viral loads, respectively (p=n.s.).

Supplementary Table S3. Sequence variants of the Rev-RI8 epitope within different HIV-1 subtypes.

HIV-1 subtype	n	RQRQIRSI	HIV-1 subtype	n	RQRQIRSI
	5	-----D--		4	-----H--
A	2	-----H--	D	2	-----H-L
	2	--N--D--		1	G----H--
	2	-----D-L		1	-----N--
	1	--N--D-L		1	------T-
	1	--Q--D--		1	---HLH--
	1	---H-S--			
	1	----LN--			
	1	-----H--		2	------A-
B	1	---R-QE-	AE	2	------E-
	1	-----QTV		1	-----H--
	1	---W--E-		1	----V-AL
	1	------DL		1	------AL
	1	-----QT-		1	------D-
	1	-----Q-V		1	Q--R-HAL
	1	--------		1	-----SA-
	1	K--H--ML			
	1	-----K--			
	1	------A-			
	1	---H----			
	1	-----Q--			
	1	------N-			
	1	---R--T-			
	6	-----H--		2	------A-
C	2	--K--H--	AG	1	--K---T-
	1	--K----L		1	-----HT-
	1	---E-S--		1	-----SA-
	1	--------		1	-----CA-
	1	K-Q-LH--		1	---H--E-
	1	------E-		1	------AL
	1	-----HTL		1	-----HE-
	1	QPKKTHLF			
	4	-----H--		4	------A-
G	2	-----N--	F	3	------E-
	2	--------		2	----V-E-
	1	------E-		1	------AL

Indicated are Rev-RI8 epitope sequences from different HIV-1 subtypes obtained from the Los Alamos HIV Sequence Compendium 2019 [19]. Dashes indicated identical amino acids as in the Rev-RI8 epitope. Amino acids letters indicate the sequence variation within the Rev-RI8 epitope. n= frequency of the indicated sequences as presented in the sequence compendium.

Supplementary Table S4. Analysis of sequence variation within the Rev-RI8 epitope.

HIV-1 subtype	n	**R**	**Q**	**R**	**Q**	**I**	**R**	**S**	**I**
A	15	0	0	4	1	1	15	0	3
B	15	1	0	0	5	0	7	9	4
C	15	2	1	5	2	2	12	3	3
D	10	1	0	0	1	1	9	1	2
F	10	0	0	0	0	2	0	10	1
G	9	0	0	0	0	0	6	1	0
01-AE	10	1	0	0	1	1	3	9	3
02-AG	9	0	0	1	1	0	4	9	1

Indicated are the number of amino acid variations within the different HIV-1 subtypes for each amino acid position of the Rev-RI8 epitope. The sequences were obtained from the Los Alamos HIV Sequence compendium 2019 [19]. n= number of sequences for each subtype.
